# Commentary: ‘Critical illness subclasses: all roads lead to Rome’

**DOI:** 10.1186/s13054-022-04265-w

**Published:** 2022-12-14

**Authors:** Mihir R. Atreya, L. Nelson Sanchez-Pinto, Rishikesan Kamaleswaran

**Affiliations:** 1 Department of Pediatrics, Cincinnati Children’s Hospital Medical Center, University of Cincinnati College of Medicine, 3333 Burnet Avenue, Cincinnati, OH 45229 USA; 2 Departments of Pediatrics and Preventive Medicine, Ann & Robert H. Lurie Children’s Hospital of Chicago, Northwestern University Feinberg School of Medicine, Chicago, IL 60611 USA; 3 Departments of Biomedical Informatics and Biomedical Engineering, Emory University School of Medicine, Georgia Institute of Technology, Atlanta, GA 30322 USA

Critical illness syndromes including sepsis and acute respiratory distress syndrome (ARDS) are major causes of morbidity among adults and children admitted to intensive care units (ICU) across the world. Clinical and biological heterogeneity among patients have, however, long confounded the identification of efficacious therapeutics, and have likely contributed to the repeated failure of clinical trials [1]. Accordingly, identification of richly phenotyped and reproducible disease subtypes through existing observational or interventional studies, development of pragmatic strategies for real-time identification of disease subtypes, and determination of heterogeneity of treatment effect (HTE) of therapeutic interventions have been emphasized as the path forward to achieving precision medicine in critical care [2].

Qin et al. recently published in *Critical Care* [3] on the identification of four pediatric sepsis subclasses through retrospective analyses of clinical and laboratory data collected within 24 h of ICU admission among 404 children enrolled in the Pediatric Sepsis-induced Multiple Organ Failure (PHENOMS) [4] study. The strengths of the study include: (1) the use of an unbiased data-driven approach to derive subclasses that demonstrate differences in clinical outcomes using k-means clustering; (2) correlative biomarker data to demonstrate biological differences between groups; and (3) proof-of-concept data suggestive of HTE when testing 14 anti-inflammatory therapies and mechanical interventions—individually and in combinations.

Among the four sepsis subclasses identified, PedSep D (*n* = 56) had the highest mortality (34%) followed by PedSep B and C which had comparable rates of death ~ 10–12%; PedSep A had the lowest mortality rate (2%). The hallmark of PedSep D subclass was the preponderance of patients with multiple organ dysfunction syndrome (MODS). In comparison, PedSep B was characterized by severe respiratory failure and central nervous system dysfunction, and PedSep C was composed of patients with predominant cardiovascular dysfunction and low rates of intubation. Biomarkers of systemic inflammation and endothelial activation demonstrated differences across the subclasses. Relative to PedSep A, patients belonging to PedSep D were characterized by increases in concentrations of interleukin (IL)-8, IL-6 and decrease in concentrations of a disintegrin and metalloproteinase with a thrombospondin (ADAMTS) 13 and low platelet counts. The differences between PedSep B and C were more subtle. Finally, non-randomized administration of corticosteroids was associated with lower mortality among PedSep D subclass, but no difference and higher mortality was observed among patients belonging to PedSep B and C, respectively.

The work detailed by Qin and colleagues represents another precision medicine tool in our armamentarium to sift through heterogeneous populations of critically ill patients. First, the subclasses identified could facilitate prognostic enrichment, i.e., identification of patients at high risk of an outcome of interest such as death and/or MODS. The biomarker profile correlated with hyper-inflammatory PedSep D subclass appears, in part, to be driven by IL-8, endothelial activation and associated low platelet counts. This bears resemblance with the prospectively validated Pediatric Sepsis Biomarker Risk Model (PERSEVERE) used to estimate ICU mortality detailed by Wong et al. [5] and a more recent iteration published in *Critical Care* to estimate the risk of MODS [6]. Similarly, there is a striking similarity of biomarkers correlated with PedSep D and those among patients with a ‘hyper-inflammatory’ phenotype of ARDS detailed by Calfee et al. [7], a subclass reproduced among pediatric patients [8], and associated with worse outcomes.

Second, the approach detailed by Qin et al. may also facilitate predictive enrichment, i.e., identification of patients subclasses with shared biological pathways that make them more susceptible to respond to a given therapy. The authors highlight the overlap of phenotypes with those detailed by our group [9], as well as sepsis endotypes among adults. Of considerable interest, the differential response to steroids demonstrated among PedSep B and C relative to those belonging to subclass D mirror findings among pediatric septic shock endotypes identified by Wong and colleagues [10], wherein patients belonging to endotype A were observed to have repression of the adaptive immune system and glucocorticoid signaling, and worse outcomes with receipt of non-randomized adjuvant corticosteroids.

The authors acknowledge the limitations of the study, including the retrospective nature of the study, relatively small sample size, and lack of a validation cohort—all of which are endemic to studies focused on critically ill children. In addition, patients belonging to PedSep D were demonstrated to represent various clinical phenotypes previously detailed by Carcillo and colleagues [11]. Thus, it is likely that the biological correlates associated with this subclass represent an average of those values across the entire subclass. It is, however, plausible that there may exist considerable heterogeneity within PedSep D. Accordingly, the interaction with therapeutic interventions demonstrated may be variable across latent subphenotypes that may exist within this subclass. Future work in cohorts enriched for those with MODS may shed light on this important question. Finally, it is worth noting that clustering algorithms like k-means may be susceptible to instability with changing the initiation seed of the clustering and the number of imputation runs, as previously demonstrated [12]. This may be particularly problematic when the sample size is limited and begets the need for external validation prior to implementation.

In summary, these observations suggest that biologically speaking, more is common across critical illness syndromes including sepsis and ARDS across the host developmental age spectrum than features that distinguish them [1]. Accordingly, research to decipher these ‘treatable traits’ through the identification of stable, reproducible, and generalizable subclasses is the need of the hour. We propose the following strategies to advance the science of precision medicine, as illustrated in Fig. 1: (1) deep ‘endo-phenotyping’ of patients at scale through integration of electronic health record data, clinical and data-driven phenotypes, and endotypes across critical illness syndromes enabled by large, multicenter collaborations; (2) leveraging high-throughput multi-omic approaches to comprehensively characterize subclass-specific biological pathways; (3) incorporating temporal dynamics across multi-modal data streams including physiological and multi-omics, acknowledging the dynamicity of critical illness, and to test functional responses to therapeutic interventions; (4) standardizing and validating methodological approaches including machine learning algorithms to reliably identify patient subclasses; and (5) developing point-of-care assays and parsimonious models to rapidly classify patients in real time in order to facilitate the design and evaluation of precision medicine strategies in future clinical trials.

**Fig. 1:**
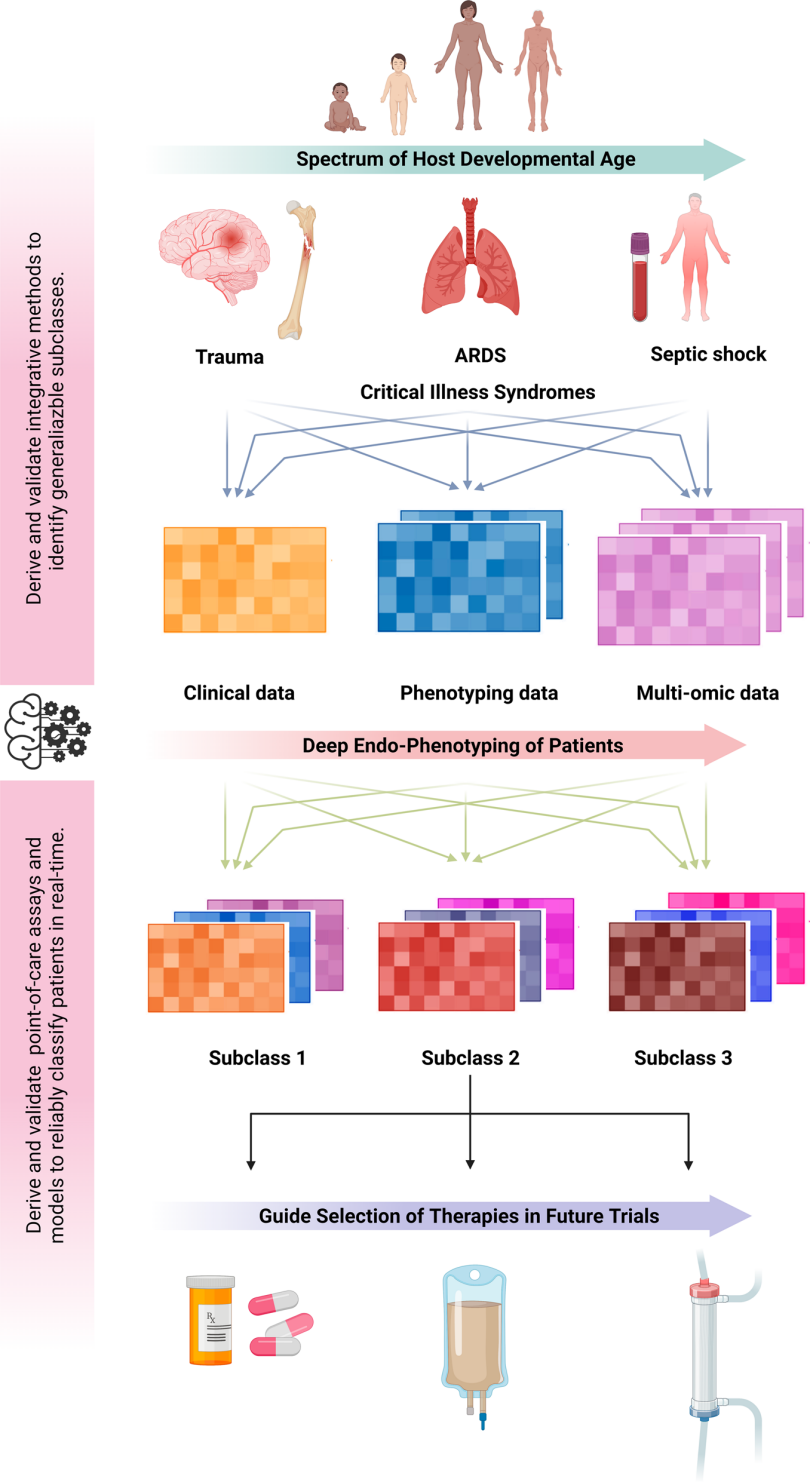
Conceptual model for identification of stable, reproducible, and generalizable subclasses across the host developmental age spectrum in critical illness syndromes. The central role for translational bioinformaticians—with expertise in computational biology and clinical informatics—to help achieve such precision in critical illness is shown on the left-hand side of the illustration. *ARDS Acute Respiratory Distress Syndrome

## Data Availability

Not applicable.
